# The Use of Allogeneic Mesenchymal Stem Cells in Childhood Steroid-Resistant Acute Graft-Versus-Host Disease: A Retrospective Study of a Single-Center Experience

**DOI:** 10.4274/tjh.galenos.2019.2019.0090

**Published:** 2019-08-02

**Authors:** Ceyhun Bozkurt, Erdal Karaöz, Başak Adaklı Aksoy, Selime Aydoğdu, Tunç Fışgın

**Affiliations:** 1 İstinye University Faculty of Medicine, Department of Pediatrics, İstanbul, Turkey; 2 Altınbaş University Faculty of Medicine, Bahçelievler Medical Park Hospital Pediatric Bone Marrow Transplantation Unit, İstanbul, Turkey; 3 İstinye University Faculty of Medicine, Department of Histology-Embryology, İstanbul, Turkey; 4 İstinye University Faculty of Medicine, Stem Cell and Tissue Engineering Research and Application Center, İstanbul, Turkey; 5 Liv Hospital, Regenerative Medicine, Stem Cell Production Center, İstanbul, Turkey

**Keywords:** Childhood, Stem cell transplantation, Steroid-resistant acute graft-versus-host disease, Mesenchymal stem cell

## Abstract

**Objective:**

Steroid-resistant acute graft-versus-host disease (srAGVHD) is the most important cause of morbidity and mortality after allogeneic stem cell transplantation. There are several treatment methods available, including mesenchymal stem cell (MSC) application. The aim of this study was to evaluate the results of MSC therapy performed in children with srAGVHD.

**Materials and Methods:**

MSC therapy was used in our center between November 2014 and December 2017 for 22 patients who developed srAGVHD. The patients were retrospectively evaluated in terms of treatment response and survival.

**Results:**

After application of MSCs, complete response was obtained in 45.5% of the subjects, partial response was obtained in 13.6%, and no response was obtained in 40.9%. We found that 45.5% of the patients were alive and 54.5% had died and our treatment results were similar to those in the literature. Response to MSC treatment was found to be the only prognostic marker affecting mortality.

**Conclusion:**

MSC application is a treatment method that can be used safely together with other treatment methods in srAGVHD, a condition that has a high mortality rate. There are almost no acute side effects. There are also no serious long-term side effects in the literature. Prospective randomized studies are required to obtain high-quality data.

## Abstract (tr)

**Amaç:**

Steroid dirençli akut graft versus host hastalığı (sdAGVHH), allojenik kök hücre naklinden sonra en önemli morbidite ve mortalite nedenidir. Mezenkimal kök hücre (MKH) uygulaması da dahil olmak üzere çeşitli tedavi yöntemleri mevcuttur. Bu çalışmanın amacı, sdAGVHH’li çocuklarda yapılan MKH tedavisi sonuçlarını değerlendirmektir.

**Gereç ve Yöntemler:**

Merkezimizde sdAGVHH gelişen 22 hasta için Kasım 2014 - Aralık 2017 tarihleri arasında MKH tedavisi uygulandı. Hastalar tedaviye yanıt ve sağkalım yönünden retrospektif olarak değerlendirildi.

**Bulgular:**

MKH uygulanmasından sonra, deneklerin %45,5’inde tam cevap, %13,6’sında kısmi yanıt alınmıştır. Deneklerin %40,9’unda yanıt alınamamıştır. Hastaların %45,5’inin hayatta olduğunu ve %54,5’inin öldüğünü ve tedavi sonuçlarımızın literatürle benzer olduğunu bulduk. MKH tedavisine yanıt mortaliteyi etkileyen tek prognostik belirteç olarak bulundu.

**Sonuç:**

MKH uygulaması, mortalite oranı yüksek bir durum olan sdAGVHH’de diğer tedavi yöntemleriyle birlikte güvenle kullanılabilecek bir tedavi yöntemidir. Neredeyse hiçbir akut yan etkisi yoktur. Ayrıca literatürde uzun vadeli ciddi bir yan etkisi yoktur. Prospektif randomize çalışmalar, yüksek kaliteli veri elde etmek için gereklidir.

## Introduction

Steroid-resistant acute graft-versus-host disease (srAGVHD) is the most important cause of morbidity and mortality developing after allogeneic stem cell transplantation. The mortality rate in srAGVHD can reach 90%. Treatment methods such as the use of various types of immunosuppressive agents, extracorporeal photopheresis (ECP), and mesenchymal stem cells (MSCs) are being attempted as second-line treatments in srAGVHD. Various rates of success have been reported with these treatment methods. The use of MSCs derived from humans has been initiated in recent years and there is an increase in the number of publications reporting that the use of MSCs is effective in srAGVHD. The mechanisms of action could be as follows: MSCs are involved in immunosuppressive and trophic immune regulation by secreting various growth factors and cytokines and by their cell-cell interaction mechanisms. Recent studies have shown that MSCs remain in the circulation for a very short time, but they are effective through immunomodulation or inhibition of T-cell activation via the exosomes they secrete, and also by influencing the tryptophan metabolism with indoleamine 2,3-dioxygenase, one of the degradation metabolites of these cells, influencing the adenosine receptor signal system of ectonucleotidase enzymes. They also act by inhibiting the immunomodulatory prostaglandins, cytokines such as IL10 and IL7, chemokines such as chemokine ligand 9, and growth factors such as transforming growth factor via programmed death receptor 1-2 (PDR 1-2) [1,2,3,4,5].

The aim of this study was to evaluate the results of MSC application in patients who developed srAGVHD that could not be controlled with other methods used in our clinic.

## Materials and Methods

The files of 22 patients diagnosed with srAGVHD who had undergone allogeneic MSC administration under suitable conditions with the approval of the ethics committee and the Ministry of Health of Turkey between November 2014 and December 2017 at the Altınbaş University Faculty of Medicine’s Bahçelievler Medical Park Hospital, Children’s Bone Marrow Transplantation Unit, were analyzed retrospectively. Assessment of AGVHD was performed according to the previously published international criteria [6]. Prednisolone or methyl-prednisolone treatment (2 mg/kg/day) was initiated for patients who had clinical manifestations of AGVHD.

Progression in one of the clinical symptoms in the first 3 days after this treatment was initiated or absence of response to the treatment within 7 days was defined as srAGVHD. Secondary treatment modalities included the addition of a new drug such as mycophenolate mofetil or sirolimus and performing an ECP procedure. An ECP procedure was performed for 17 subjects for a total of 4 times on 2 consecutive days with an interval of 1 week. MSCs were administered to the subjects who did not respond immediately after the 4^th^ ECP procedure. MSCs were administered to all patients in the form of an intravenous infusion within 1 h in isotonic saline at a standard dose of 2 million/kg for a minimum of 2 doses and a maximum of 4 doses according to the clinical response observed, with an interval of 1 week. The median duration between the diagnosis of AGVHD and initiation of MSC therapy was 15 days (range: 6-55).

Ten patients received 2 doses, 4 patients received 3 doses, and 8 patients received 4 doses. The median dose of MSCs was 3x10^6^ cells per kilogram of body weight. Complete response (CR) to treatment was defined as improvement of all symptoms. Partial response (PR) was defined as improvement of clinical symptoms without complete disappearance. No response was defined as absence of response in clinical symptoms or worsening of the clinical picture. Evaluation of the patients’ responses to MSC treatment was performed 28 days after the first infusion and evaluation of survival was performed at least 6 months after the first infusion. In accordance with the Declaration of Helsinki, informed consent was obtained from the families of the patients who were administered MSCs. Approval was also obtained from the İstinye University Faculty of Medicine’s Ethics Committee for this study with approval number (2017-KAEK-120)/51.

### Procedure for Preparing Mesenchymal Stem Cells

Umbilical cord tissue-derived MSCs manufactured under current good manufacturing practice (cGMP) conditions (LivMedCell, İstanbul, Turkey) were used in this study. Human umbilical cords were obtained from healthy donors with their written approval. Each umbilical cord unit was manipulated under sterile conditions. These units were cut into sections of approximately 5 cm. The parts were washed with DPBS solution to remove the blood. The arteries and veins were removed to avoid endothelial cell contamination. Wharton’s jelly sections were then divided into smaller pieces. Tissue explants were placed into 100-mm2 cell culture plates and cultured in the Nutristem cell culture medium supplemented with 2% human serum and 50 U/mL penicillin-streptomycin. MSCs were grown in a humid atmosphere containing 5% CO_2_ at 37 °C. The cells were subcultured to the third passage. Cell preparation steps were performed according to cGMP requirements as described previously [7,8,9]. The cells were characterized by identifying the potential for differentiation using a flow cytometer and immunohistochemical analysis, cell aging, cell cycle, annexin V/PI staining, and telomerase enzyme activity at the third passage. Quality control and quality assurance for the production of these cells were conducted in accordance with the standards of the Turkish Pharmaceuticals and Medical Devices Agency (TMMDA).

### Statistical Analysis

IBM SPSS Statistics 22 (IBM SPSS, Turkey) was used for the statistical analyses. While evaluating the study data, the compliance of quantitative data with a normal distribution was evaluated with the Shapiro-Wilks test and it was found that the parameters did not show a normal distribution. In addition to descriptive statistical methods (mean, standard deviation, frequency), the Kruskal-Wallis test was used for comparison of age for response and the Mann-Whitney U test was used for comparison of age for outcome when comparing quantitative data. Fisher’s exact chi-square test and the Fisher-Freeman-Halton test were used for comparison of the quantitative data. A p-value of <0.05 was considered statistically significant.

## Results

A total of 22 subjects who developed srAGVHD following allogeneic stem cell transplantation between November 2014 and December 2017 were evaluated. The study was conducted with children aged 15 to 204 months. The study group consisted of 10 (45.5%) male and 12 (54.5%) female subjects. The mean age of the children was 88.95±61.82 months and the median age was 66 months. Table 1 presents the transplantation diagnosis and transplantation process data for these patients.

Malignancy was present in 41% of the children. The stem cell source was peripheral blood (PB) in 50%, bone marrow (BM) in 40.9%, and PB+BM in 9.1%. The donor source for transplantation was a matched unrelated donor (MUD) in 54.5% cases and matched related donor (MRD) in 40.9%. Haploidentical transplantation was performed for only 1 child. A myeloablative regimen was administered for preparation in 86.4% of the children. ECP was performed for 77.3% of the subjects who developed AGVHD. When the subjects were graded according to their pre-treatment AGVHD status, it was found that 18.2% had Grade 2 AGVHD, 45.5% Grade 3 AGVHD, and 36.4% Grade 4 AGVHD.

After administration of MSCs, CR was obtained in 45.5% of the subjects, PR was obtained in 13.6%, and no response was obtained in 40.9%. Table 2 presents the overall and organ-specific AGVHD grades and the general response to MSC therapy. When the patients were evaluated according to organ-specific response, a 42% response rate was obtained in the liver AGVHD group, a 77% response rate was obtained in the skin AGVHD group, and a 44% response rate was obtained in the gastrointestinal AGVHD group. When the final status was evaluated, it was found that 45.5% of the patients were alive and 54.5% had died.

When the deceased subjects were evaluated, it was found that 40% of the male subjects and 66.7% of the female subjects had died. The difference was not statistically significant (p>0.05). There was also no statistically significant difference between the mean ages of the children who had died and those who were alive (p>0.05).

We found that 44.4% of the children diagnosed with a malignancy and 61.5% of the children who had a non-malignant disorder had died. The difference was not statistically significant (p>0.05).

The mortality rate was found to be 36.4% in the children whose stem cell source was PB, 77.8% in the children whose stem cell source was BM, and 50% in the children whose stem cell source was PB+BM. The difference was not statistically significant (p>0.05). Similarly, the mortality rate was found to be 66.7% in the children whose donor type was MRD and 50% in those whose donor type was MUD. Again, the difference was not statistically significant (p>0.05).

The mortality rate was found to be 47.4% in the children with a myeloablative conditioning regime and 53.6% in those with a non-myeloablative conditioning regimen. The difference was not statistically significant (p>0.05).

The mortality rate was found to be 67.4% in the children who had undergone ECP and 20% in the children who had not undergone ECP. The difference was not statistically significant (p>0.05).

The mortality rates by grade of AGVHD were found to be 50%, 50%, and 62.5% for Grades 2, 3, and 4, respectively. The difference was not statistically significant (p>0.05).

A statistically significant correlation was found between treatment response and mortality (p=0.001; p<0.05). Mortality was observed in all patients who did not respond to MSC treatment (100%), in 66.7% of those with PR, and only in 10% of those with CR. A statistically significant correlation was found between response to MSC treatment and mortality based on these results (p<0.001; p<0.05).

Table 3 presents the factors affecting survival in our patients.

We did not observe any side effects related to MSC infusion in any of the patients.

## Discussion

The use of MSCs in srAGVHD has gradually increased since 2004 when they were clinically used for the first time. Although it has been reported that prophylactic use of MSCs before stem cell application decreases the AGVHD rate [10,11,12], MSC therapy is usually used after AGVHD is diagnosed. It has been reported that the response rate is 15%-75% [13,14,15,16,17]. The response was reported to be better in cases of childhood srAGVHD in studies that evaluated pediatric and adult cases together [16,18]. The response rate in our series was approximately 58%, comparable to the literature.

There are various applications regarding the MSC donor source. The usual MSC source is bone marrow [19,20]. We obtained MSCs from Wharton’s jelly derived from the cord blood of a single donor. Kuçi et al. [21] reported an overall survival rate of 71±11% at 2 years of follow-up for their entire patient cohort with MSCs that they prepared from monocytes from multiple donors compared to a survival rate of 51.4±9.0% in their historical control group. They stated that the reason could be allosuppression differences that might have been present between the MSCs obtained from the donors, and they believed that they could increase the mean allosuppression rate in MSC treatments by increasing donor diversity. Randomized prospective studies are required to determine the effectiveness of MSCs obtained from single or multiple donors.

The frequency and number of infusions for MSC applications can vary. The reported number of MSC doses ranges from 1 to 7 and the doses range from 0.4x10^6^/kg to 10x10^6^/kg [19]. Kurtzberg et al. [14] administered MSC treatment in pediatric cases of srAGVHD for a consecutive 4-week period at a dose of 2 million/kg (the same dose as in our study) twice a week. The general response rate was 61.3%. This response rate is similar to ours. Evaluation of these two studies revealed that application of MSC treatment twice a week had no additional benefit and increased treatment costs. Similar results were obtained with MSC application in cases of srAGVHD using intervals of 2 weeks with a different method in the study of Erbey et al. [22]. MSC applications are generally conducted with intervals of 1 week according to the literature.

When the factors affecting survival in MSC treatments were investigated in our study, the presence of a response to MSC treatment was the only prognostic indicator affecting mortality. The general survival rate was found to be 63.8% in patients with CR to MSC treatment in the 2^nd^ year following MSC application, whereas it was found to be 0% in the groups with PR or no response in the study conducted by Erbey et al. [22] in Turkey. Similarly, the general survival rate was found to be 69% in patients with CR to treatment, whereas it was found to be 0% during the 2.9-year follow-up period following MSC administration in srAGVHD patients in the study by Ball et al. [23]. In the study conducted by Resnick et al. [18], multivariate analysis demonstrated that initial response (partial or complete) had a significant independent influence on 6-month survival (hazard ratio: 29.4). These findings also support our results showing a high survival rate with MSC treatment when CR was obtained.

Introna et al. [16] reported better response in Grade 2 subjects compared to Grade 3 and 4 subjects in their study. Resnick et al. [18] reported that the overall survival was lower in Grade 4 GVHD patients compared to Grade 2 and 3 GVHD patients. No association was observed between grade status and treatment response in our patients.

It has been proposed that tumor recurrence [24,25] and an increase in infections may occur as a long-term side effect of MSC applications. No short-term acute side effects were observed in relation to the MSC applications in our study. Disease relapse was observed in one of the 9 patients who had malignancy in our study. Kuçi et al. [21] found that the relapse rate was 9% in their patients who had srAGVHD. We did not evaluate whether the infection rate had increased. Other studies have not reported an adverse effect that increased the rate of infection [12,26,27].

In a great portion of our subjects, ECP was applied before MSC administration. The weakness of our study was thus that the responses were not solely associated with MSC administration. There was a possibility that the ECP procedure also contributed to this improvement. MSC application might have increased the immunosuppressive effect of ECP or might have possibly led to an improvement in GVHD by itself.

## Conclusion

MSC administration is a treatment method that can be used safely together with other treatment methods in srAGVHD, a condition that has a high mortality rate. There are almost no acute side effects. The literature also reports no serious long-term side effects. Randomized prospective studies are required to obtain high-quality data about the effectiveness, safety, and side effects of MSC application in cases of srAGVHD.

## Figures and Tables

**Table 1:**
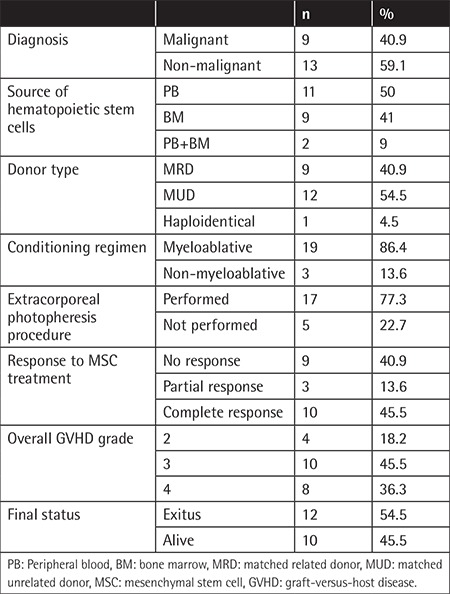
Patient and transplantation parameters.

**Table 2:**
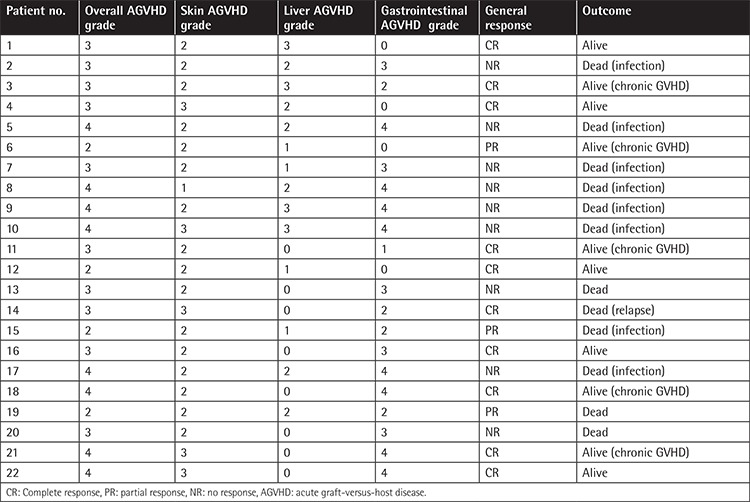
Overall and organ-specific acute graft-versus-host disease grades and response to mesenchymal stem cell therapy.

**Table 3:**
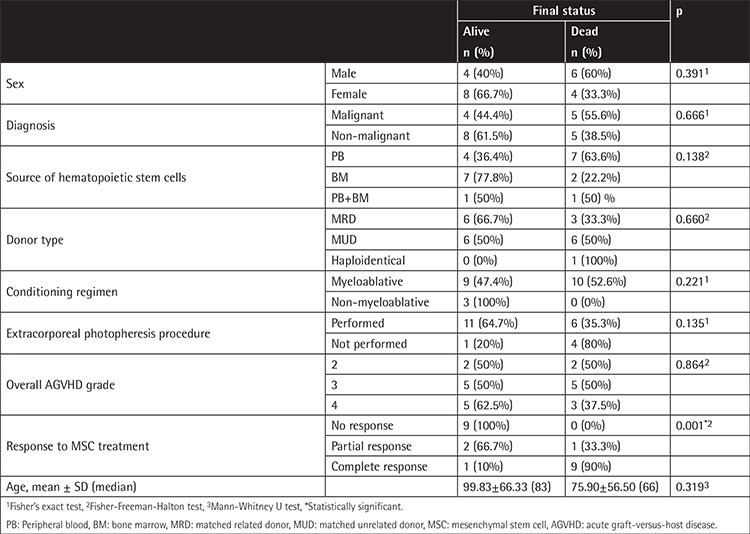
Evaluation of the parameters affecting final status.
